# Unmasking Subglottic Stenosis in an Asthmatic Emergency Needing Front-of-Neck Access: A Case Report

**DOI:** 10.7759/cureus.74847

**Published:** 2024-11-30

**Authors:** Yun Hao Leong, Victoria Yu Jia Tay, Henry Wenjie Chua

**Affiliations:** 1 Pain Management, Singapore General Hospital, Singapore, SGP; 2 Anesthesiology, Singapore General Hospital, Singapore, SGP; 3 Anesthesiology, Sengkang General Hospital, Singapore, SGP

**Keywords:** asthma, diagnostic delay, respiratory failure, subglottic stenosis, tracheostomy

## Abstract

Subglottic stenosis poses a rare but life-threatening risk for difficult tracheal intubation. Here, we report a unique case of undiagnosed subglottic stenosis discovered during emergency intubation of an 80-year-old woman with type 2 respiratory failure from infective exacerbation of asthma. A small calibre size 5.0 tracheal tube was successfully inserted, but significant difficulties with mechanical ventilation, nebulizations, and secretion management necessitated front-of-neck access with a surgical tracheostomy. Drawing on our experience of this case, we consider the diagnostic and management challenges of subglottic stenosis and discuss the issues of mechanical ventilation through small calibre tubes in patients with severe bronchospasm.

## Introduction

Subglottic stenosis (SGS) refers to a condition in which the airway is narrowed inferior to the glottis [1]. This condition is rare but poses significant risks, particularly by complicating airway management and making tracheal intubation challenging, even life-threatening, in emergency settings. The most common cause of SGS is iatrogenic, with previous intubation or tracheostomy contributing to about 50% of cases [2,3]. However, it can also be idiopathic in nature or due to less common causes like inhalational burns, irradiation, infections such as bacterial tracheitis, and collagen vascular diseases [4]. 

Patients with SGS often present with respiratory symptoms such as wheezing, shortness of breath, and airway compromise, which can mimic more common conditions like asthma [1]. This symptomatic overlap frequently leads to misdiagnoses and delayed identification, with some cases remaining undetected for years [5]. A small number of published case reports have described undiagnosed SGS discovered after induction of anesthesia in an elective setting [6]. Here, we report a unique case of undiagnosed SGS in an asthmatic patient who required emergency intubation and eventual tracheostomy for severe exacerbation of asthma.

## Case presentation

An 80-year-old woman, with a height of 144 cm, a weight of 63.5 kg, and a body mass index of 30.6 kg/m^2^ presented with severe respiratory distress due to an exacerbation of asthma from metapneumovirus pneumonia. She has a known history of severe asthma on long-term salmeterol 25 mcg/fluticasone 250 mcg (two puffs twice a day) and tiotropium 2.5 mcg (two puffs once a day) inhalers, as well as oral montelukast 10 mg (once every night). Nine years ago, she required intubation and two days of mechanical ventilation for an episode of severe asthma exacerbation. She was extubated and had no known records of previous tracheostomies or difficult airways.

Despite initial treatment with salbutamol/ipratropium nebulizations (1 ml salbutamol, 2 ml ipratropium, mixed with 1 ml of 0.9% sodium chloride to a total volume of 4 mls, given every 20 minutes), intravenous (IV) hydrocortisone (200mg followed by 100mg every eight hours), and IV magnesium sulfate (2.5 g infusion over one hour), the patient deteriorated clinically in the ward. Arterial blood gas analysis showed severe type 2 respiratory failure (pH of 7.332, PaCO2 of 75.6 mmHg, and PaO2 of 130.2 mmHg while receiving 2L/min of supplemental oxygen via nasal prongs), and she was transferred to the intensive care unit (ICU) for intubation.

Pre-intubation airway examination was unremarkable. A rapid sequence induction was performed using IV propofol 30 mg, ketamine 30 mg, fentanyl 50 mcg, and succinylcholine 100 mg. A C-MAC® video laryngoscope with a size three Macintosh blade revealed a grade-one glottic view. Insertion of a size 7.5 portex tracheal tube with a pre-form stylet was attempted, but only the tip of the tube could be advanced past the vocal cords due to significant resistance on insertion. A bougie was then inserted successfully, but both size 7.0 and 6.5 tubes could not be railroaded past the vocal cords due to resistance on insertion. Intubation attempts were paused for bag-mask ventilation to maintain the patient’s oxygen saturation>95%. A second anesthetist was called for assistance, and a flexible bronchoscopy was performed to evaluate the cause of the obstruction. The patient was given additional IV propofol 90 mg, ketamine 20 mg, rocuronium 50 mg, and fentanyl 50mcg prior to subsequent airway manipulation. A 3.8 mm Ambu® aScope™ was passed successfully beyond the vocal cords and revealed a segment of severe circumferential subglottic stenosis. Attempts to intubate with a size 6.0 portex tracheal tube were unsuccessful and a size 5.0 tube was eventually inserted.

Volume control ventilation was started on the following settings: FiO2 0.4, tidal volume (TV) 200 ml, positive end-expiratory pressure (PEEP) 0 mmHg, and respiratory rate (RR) of 18 breaths per minute. IV propofol (30 mg per hour), fentanyl (80 mcg per hour) infusions, and paralytic agents were given to improve ventilator synchrony. Despite this, significant difficulties were encountered in achieving sufficient minute ventilation. Attempts at increasing tidal volume or respiratory rate led to significant increases in peak inspiratory pressures as well as the development of auto-PEEP. The need for repeat nebulization of bronchodilators through the tube as well as secretions further compounded this issue. Otolaryngology was consulted for surgical tracheostomy for a larger calibre definitive airway, and a cuffed, non-fenestrated size 7.0 Portex® Blue Line ULTRA® Suctionaid® tracheostomy tube was inserted below the level of the subglottic stenosis lesion. The insertion was done in the operating room with no complications.

**Figure 1:**
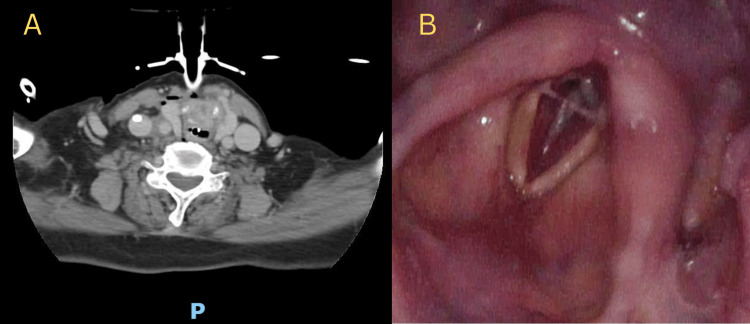
Axial view of contrasted neck CT scan (A) and bronchoscopy image at vocal cord level (B) showing severe subglottic stenosis with posterior glottis gap of about 2 mm

Subsequent computed tomography (CT) scans of the neck revealed severe luminal stenosis of the glottic and subglottic larynx with symmetrical soft tissue thickening (Figure 1A). There was no evidence of enhancing lesions or any discrete laryngeal mass. The cause of the stenosis was attributed to a possible iatrogenic cause (from previous intubation) or idiopathic. Nasoendoscopy revealed a 90% subglottic stenosis with a small posterior glottic gap (Figure 1B). The patient was eventually weaned off the mechanical ventilator 5 days later and discharged from the ICU. Written consent for the publication was then obtained from the patient after her discharge from the ICU.

## Discussion

Diagnostic challenges of subglottic stenosis

Due to the narrowing of the upper airway, SGS typically presents with stridor, dyspnea, and chronic cough, all of which are symptoms that can lead to a misdiagnosis of asthma or chronic obstructive pulmonary disease.

Previous case reports have described cases of SGS mimicking asthma [5]. In these cases, patients are typically started on asthmatic medications with little to no improvement in their condition. Methods proposed to differentiate the two conditions include the use of serological testing for anti-neutrophil cytoplasmic antibodies (ANCA), angiotensin-converting enzyme levels (ACE), pulmonary function testing, and computed tomography [7]. 

However, in this case, the patient had a long history of asthma that had been responding to typical asthma therapy. In addition, she also had a previous intubation event for severe asthma that did not reveal any airway abnormalities. The emergent nature of the intubation also precluded the possibility of pre-intubation testing. This unusual presentation emphasizes the importance of recognizing the diagnostic challenges associated with subglottic stenosis. An acute awareness of potential cognitive biases due to a patient’s previous medical history is crucial for clinicians to avoid diagnostic delays and poor outcomes in emergency airway management.

Application of difficult airway algorithms

Failed intubation or unanticipated difficult intubation can lead to severe complications, including death, hypoxic brain injury, or emergency surgical airways. Management algorithms like the ASA Difficult Airway Algorithm [8] and the Difficult Airway Society (DAS) 2015 guidelines [9] have been developed to provide simplified, evidence-based guidance for clinicians to respond to such situations in an expedient manner and have been widely used in clinical practice.

However, it is important to remember that nuances in a case might require clinicians to exhibit critical thinking and adaptive application of such guidelines. For example, based on the DAS guidelines, repeated failure of tracheal intubation should be followed by an attempt to insert a supraglottic device for oxygenation. However, the severe bronchospasm and high airway pressures in this patient would have made ventilation through a supraglottic device extremely difficult and might not have been an appropriate next step in the patient’s airway management. As the main difficulty was due to an obstruction of unknown cause beyond the vocal cords, a decision was made to perform fiberoptic bronchoscopy for on-the-spot evaluation of the obstruction, even though this is not a step recommended in most difficult intubation algorithms. This decision confirmed the diagnosis of subglottic stenosis in this case and helped guide the decision to further downsize the tracheal tube.

Ventilatory challenges of small calibre tubes in bronchospasm

Previous studies have shown that trachea pressures and breathing work remain similar during routine anesthesia with tracheal tube sizes as small as 6.0 mm [10]. However, this may vary in conditions such as status asthmaticus, where previous studies have demonstrated an association between smaller tracheal tube sizes and incrementally higher mortality in intubated patients [11]. When smaller tube sizes are used, it is important to consider Hagen-Poiseuille's equation for laminar flow, which relates the flow rate of a fluid through a tube to its radius, length, and viscosity of the fluid. The flow rate is directly proportional to the fourth power of the radius, and thus a drop in tube radius by half will decrease the flow rate 16-fold or cause a corresponding increase in the pressure gradient. This means that a minor decrease in tracheal tube diameter will result in a significant rise in the driving pressure required to achieve the same flow rate. This effect is made worse in patients with respiratory compromise, where the increased respiratory rate and peak inspiratory flows cause a transition from laminar to turbulent flows and can further increase the resistance and pressure changes in the tube [12]. Obstruction in the lower airways from bronchospasm can further compound this effect and make successful ventilation impossible. This was observed in our patient when attempts to increase the RR and tidal volumes beyond a certain point led to significant increases in inspiratory pressures and the development of auto-PEEP. In such cases, increasing the ventilator pressure limits may be necessary to allow for adequate tidal volume ventilation. Allowing permissive hypercapnia with arterial PaCO2 to as high as 90 mmHg may also be required to balance against the cost of achieving normocapnia with the risks of hyperinflation and barotrauma [13].

In addition, the tracheal tube functions not only as a conduit for ventilation but is also required for suctioning and the delivery of key medications. Administering salbutamol nebulizations through a small calibre of the tracheal tube likely resulted in a substantial portion of nebulized salbutamol failing to reach the distal airways, thus limiting the bronchodilatory effect [14]. The administration of nebulized medications also likely caused worsening turbulent flows and contributed to the high ventilator pressures [15]. Ultimately, in this case, a multidisciplinary discussion with the otolaryngologist and ICU team led to the decision for an emergency tracheostomy for a larger calibre definitive airway. Notably, after the tracheostomy was performed, ventilation improved markedly, and the patient was eventually weaned off the ventilator and discharged from the ICU five days later.

## Conclusions

This case highlights the diagnostic challenges associated with undiagnosed subglottic stenosis (SGS), particularly in patients with a history of chronic respiratory conditions like asthma. Clinicians must maintain a high index of suspicion and recognize potential cognitive biases that could delay diagnosis, even in patients with a history of uneventful intubation. While difficult airway algorithms provide essential guidance, managing anatomical abnormalities such as subglottic stenosis often necessitates tailored approaches. Clinicians should be aware of the challenges of ventilating patients with small-calibre tubes, particularly in the setting of severe bronchospasm, and should be prepared to modify ventilatory strategies to avoid complications associated with elevated airway pressures. Furthermore, administering nebulized medications and suctioning through small tubes also requires careful consideration due to the risk of increased resistance and ineffective drug delivery. This case underscores the importance of a tailored, multidisciplinary approach to airway management in patients with complex anatomical variations.
